# Financial Crisis in Management Stress: From the Perspective of Crisis Anxiety of Others

**DOI:** 10.3389/fpsyg.2022.854746

**Published:** 2022-07-04

**Authors:** Bin Liu, Jing Zhu, Fangguo Su, Bin Wen, Yingqi Wu

**Affiliations:** ^1^School of Economics and Management, Jiangxi Science and Technology Normal University, Nanchang, China; ^2^School of Business Administration, Guangdong University of Finance and Economics, Guangzhou, China; ^3^College of Management, Shenzhen University, Shenzhen, China; ^4^School of Government, Shenzhen University, Shenzhen, China; ^5^School of International Economics Trade, Jiangxi University of Finance and Economics, Nanchang, China

**Keywords:** trait anxiety, state anxiety, others' crisis anxiety, occupational health, management stress

## Abstract

The crisis anxiety of others is a phenomenon that goes hand in hand with the spread of the occupational health pandemic. It is becoming increasingly important to better understand its emergence process, especially in the era of greater uncertainty. This study aims to examine the impact of the external financial crisis on managerial stress among financial employees. The sample consists of 347 senior managers and financial employees from companies in China. The empirical analysis shows that external financial crises have significant effects on anxiety levels, especially external corporate crisis, debt crisis and growth crisis both have mediating effect on the relationship between anxiety level and pressure management and the relationship between external financial crisis and pressure management. This study explores the rules for the emergence of anxiety among corporate managers and expands the scope of environmental factors that need to be discussed in the study of corporate financial management. This study provides theoretical implications for the psychological study of Financial Management and practical implications for corporate financial management.

## Introduction

Fueled by economic globalization and the spread of the pandemic, contemporary enterprises are facing rapidly changing market competition. Considerable research has been conducted on how to timely grasp the advantages and disadvantages of corporate products or services, and dealing with various problems and challenges is an important topic for enterprises to gain absolute advantages. Contingency theory holds that the management behavior is self-correction and adjustment to strengthen environmental adaptability (Zhou and Liu, 2013). The environment mentioned here includes not only the social, economic, and cultural external environments faced by corporations, but also their factors that are “determined” due to management models or institutional norms (Franco, 2021). At the same time, in the context of the new era of the Internet Economy, the organizations encounter challenges from internal management governance and external pressure from marketing, including disruptions, risks and uncertainties arising from economic system reform, economic freedom improvement and capital investment increase (Mia and Clarke, 1999). From the perspective of market relations, enterprises are not independent individuals, and their development in all aspects is inseparable from the reference and comparison of the same-level external enterprises with similar strategic resources under the same market environment (Wang W. et al., 2020; Gibson et al., 2021; Sun and Li, 2021). Generally, the academic research on sustainable competitive performance is more focused on financial performance, technical innovation, and corporate social responsibility (Waheed et al., 2020; Li et al., 2021a,b; Yi et al., 2022). However, limited research has been conducted on how to prevent risk and control behavior guidance for individuals (Yi et al., 2021), especially the employees in the financial industry who experience serious anxiety and health problem under the highly uncertain market situation. A large amount of practical experience shows that scarce, non-fully tradable, and hard-to-act resources of external enterprises have a significant impact on the internal management of enterprises (Severo et al., 2020), and such resources include tangible resources and intangible assets such as management, experience and modes that can produce better performance.

Existing literature on corporation risk management is largely focused on internal control-level, meanwhile, the external macro-environment is also considered to a certain extent, to improve the corporate's ability to adapting changes in the external environment. However, most of them ignore the relatively more important part of external factors, or the competing companies in the same industry, as these companies will provide rich experience and lessons in the financial crisis to cope with the ever-changing macro-environment of financial management for other corporations. While being alert to the corporate internal management, the psychological anxiety and emotional exhaustion will be inevitably brought to the middle and senior managers, therefore, causing management pressure. Specifically, when learning that external enterprises are in crisis, the corporate managers will inevitably have empathy or self-pity in the changing process with psychological pressure. This is pervasive in the current context of market competition, and the psychological problems presented when the managers face the pressure will aggravate the uncertainty and volatility of the subsequent corporate financial plans, policy choices, and decision making. Therefore, clarifying the path and mechanism of the external corporate financial crisis on the management stress and conducting follow-up stress control effectively and timely have become key factors for successful corporate management.

Okruszek et al. (2020) reports a positive relationship between crisis perception and emotional response and Yu et al. (2021) have further developed job insecurity from the negative emotions, which inspired this study. The COVID-19 pandemic has led enterprises worldwide to implement unprecedented response strategies for economic development. Both enterprises and employees are seriously affected, especially the corporate financial status and the financial personnel. Consequently, our research perspective is positioned in the external financial crisis, especially the impact on the psychological status and management behavior of financial management personnel. Thus, we designed this study to explore the causes of the crisis anxiety of others and make rational financial warnings by empirically examining the internal correlation between the external corporate financial crisis and corporate management. The finding of this study will provide theoretical implications for the psychological stress control of corporate financial employees and the empirical inter-disciplinary study of corporate management and psychology.

The remainder of this study is organized as follows. Section Literature Review and Hypotheses Development reviews the main literature and hypotheses developed based on the previous literature. Among them, we mainly introduce the causes of the external crisis and the psychological and behavioral processes that affect financial personnel, and further introduce the important roles of state anxiety and financial warning capability in this process. Section Methodology discusses our research methodology and specifically analyses the process, to examine our related hypotheses. Furthermore, we conclude with a discussion summarizing our results, propose the theoretical and managerial implications of our work, and present some of our reflections on future research.

## Literature Review and Hypotheses Development

### Crisis Anxiety of Others

In the literature on social psychology, anxiety refers to an aimless subjective feeling of an individual in the face of potential threats and dangerous environments, and that excessive anxiety can affect their well-being in normal work and daily life (McDowell et al., 2020; Zhang et al., 2020; Ali et al., 2021; Secosan et al., 2021). In general, anxiety is divided into state anxiety and trait anxiety (Charles, 1972), and the former refers to a temporary and heterogeneous emotional state or response caused by the influence of a specific situation (Liu et al., 2021), and the latter focuses more on personality traits, and it is more stable than state anxiety. The State-Trait Anxiety Inventory Scale (STAI) is commonly used to assess the levels of individual state anxiety, and it has been widely applied on empirical clinical psychology (Näslund et al., 2017), psychosomatic disease (Hallit et al., 2018), psychotherapy and counseling (Ma et al., 2013). In real life, anxiety is not only concerned in psychology but also in many other disciplines. It is found that although STAI is widely used, many studies that applied the scale may cause psychological resistance in the respondents, and thus their willingness will be affected (Schaufeli et al., 2002). Therefore, Marteau and Bekker (1992) selected the questions highly related to the scale from the presence and absence of anxiety and formed the Short State Anxiety Inventory (SSAI). The existing applications mostly focus on the impact of state anxiety on cognition, behavior, and interpersonal relations of human beings as well as countermeasures and suggestions. Numerous studies have confirmed that conflicts between peers and rejection by peers can cause individual loneliness and the longer the anxiety in this negative relationship, the greater the aggressive behavioral tendency (Dittrick et al., 2013). Later scholars identify that state anxiety negatively affects human working memory and productivity by limiting working memory capacity and interfering with the filtering of effective information (Ward et al., 2020; Deng et al., 2022; Duan et al., 2022). With more convenient information circulation, external corporate crisis brings anxiety to the corporate managers repeatedly, and such anxiety profoundly affects the corporate development and management and has a greater impact on financial work. Bearing this in mind, this study focuses on interpersonal activities and the information dissemination process to explore the causes of crisis anxiety of others.

For modern enterprises, finance is their pivot, and financial security is an important premise for them to carry out financial and operating activities continuously. In previous studies, the academia mainly paid attention to corporate financial security problems, analyzed the influences of market changes, capital structure, cash flow capacity, and capital price on corporate financial practices (Huang and Yen, 2019), and discussed the internal mechanism of learning good external corporate systems and taking warnings from poor systems. However, the existing studies ignored the transmission effect of the incident itself, that is, the crisis is of abruptness and destructiveness, causing emotional fluctuations and adverse social effects. In the context of crisis, the most significant point in the financial management work is the behavioral bias through the external corporate financial crisis, which intensively embodies the management stress and crisis anxiety of others. In this study, crisis anxiety of others has the following connotation: witnessing the occurrence of the external corporate crisis, the managers encounter a temporary and differentiated emotional state and aimless subjective feeling in the face of potential threats and a dangerous environment in the existing management work. Such subjective feelings are generally regarded as state anxiety, or management stress related to financial security issues in corporate financial management.

### External Financial Crisis and State Anxiety

When the environment is abnormal, people's minds will send out a kind of warning signal at the early stage, namely, anxiety (Han et al., 2015). In daily operation management, anxiety generally stems from the interaction of internal and external factors. The internal factors include the formulation and implementation of the internal control system, and the external ones refer to the objective environmental factors not transferrable by the will of the enterprise, such as industrial market environment, competitive corporate management mode, economic and industrial policy (Lang and Stulz, 1992), among which the influences of internal and external factors are particularly prominent in corporate financial management. Specifically, enterprises with low-quality internal control may have irrational emotions in making financial decisions, especially anxiety, panic and other negative emotions that will affect the formulation and implementation of corporate strategies (Yeh and Liao, 2020). The corporate cash flow and investment turnover may also be affected due to financial structure problems of external enterprises, falling into a debt crisis (Onsongo et al., 2020). On the other hand, industries with a poor competitive environment will disperse their market forces and monopoly profits, which will increase cash flow volatility and bankruptcy risk of the enterprises. The financial problems of the competitive enterprises in the same sector will become the “alert signal” for the whole industry to force the managers to take their internal financial problems more seriously and produce a certain degree of the crisis anxiety of others.

Low management efficiency and fierce market competition are the main internal and external causes of the corporate financial crisis (Hertzel and Officer, 2012). If the enterprise has poor operating conditions, serious debt defaults, investment problems and a continuous net outflow of cash flows (Ruan and Liu, 2021), it is more likely to have a financial crisis and take corresponding crisis response measures. After the external corporate financial crisis is exposed, the negative incident as a signal will attract the attention of the industrial investors and competitors, and in a short time, it will affect the judgment of the enterprise and the value of the whole industry. It sends a signal that the market development of the industry is depressed, which will affect the corporate market competitiveness and even the whole industrial sector. As the enterprise faces more market pressure, the managers will inevitably have the anxiety that crisis anxiety of others will affect the formulation and implementation of their management decisions (Dionne et al., 2018). Due to the high speed, strong urgency, and unpredictability of the crisis, coupled with the rapid evolution of the crisis state, the negative sentiment in the industrial sector constantly spreads and forces managers to overreact (Kalmbach et al., 2020) and make impulsive adjustments or changes to the decisions and systems related to the crisis. In corporate financial management, the key references for corporate management mostly exist in the financial relationships between enterprises and suppliers, customers, employees, and creditors. When external enterprises fall into financial crisis, these financial relationships that maintain the survival of enterprises will be damaged, resulting in indirect financial crisis costs and even secondary transmission of information that will affect the operation and management of competitive enterprises in the same industrial sector. The enterprise involvement and the external corporate financial crisis will inevitably impact on industrial development. A little carelessness may produce a series of chain reactions, bring oppressive pressure management, and affect the normal corporate operation. Thus, the present study proposes the following hypotheses:

*Hypothesis 1: The operational crisis of external enterprises has a significant positive impact on state anxiety*.
*Hypothesis 2: The debt crisis of external enterprises has a significant positive impact on state anxiety*.
*Hypothesis 3: The growth crisis of external enterprises has a significant positive impact on state anxiety*.

### State Anxiety and Management Stress

Anxiety is a common negative emotion in daily life. When people feel anxious, they tend to make bad decisions or delay decision-making, which has a strong negative impact on daily management (Gino et al., 2012). The anxiety accompanies corporate financial management, and the state anxiety is essentially the emotional reaction brought about by individual self-discrepancy. At work, the managers often compare with social standards, and self-discrepancy occurs when the ideal criteria are not met (Cheung, 1997). Such discrepancy will bring an individual a negative state, namely anxiety. As Waheed et al. (2020) assumed, the enterprise is a complex system composed of environment, managers, and employees. Furthermore, the changes in the external environment will disrupt the normal working behavior of the employees. While the changing external environment brings about increased work demand, it is bound to disrupt the normal work order of the employees.

As a negative mood, state anxiety will occupy more cognitive resources of employees and consume limited self-control resources. Previous studies have adopted this theory from different perspectives. For example, Gonzalez and Winkler (2019) confirmed in their study that when a crisis occurs, individuals first make cognitive judgments of various incidents. Due to various complicated situations and all-inclusive information, people's understanding of the incident is affected by multiple factors, and the cognitive judgment is usually one-sided. Lwin et al. (2020) further inferred that this one-sided cognition will cause people to refuse to face the reality, produce negative emotions and passively adapt to the current situation, and thus anxiety will be aroused. From the perspective of individuals, Norris et al. (2020) found that the occurrence and development of a financial crisis are usually accompanied by some related issues, which will easily cause the individuals in similar situations or with the same emotions to “put themselves in others' position”. This will lead the existing negative emotions deep in their brain to affect the judgment of the incident itself through the “historical memory”. Hannah et al. (2021) report that the crisis is not just a simple accidental incident, and the management diagnosis and operational risks it triggers are the “clusters” of emotions and contradictions, which may lead to the corporate trust crisis or major management stress driven by various complex environments and conflicts of interest. Accordingly, the present study makes the following hypothesis:

*Hypothesis 4: State anxiety has a significant positive effect on management stress*.

When a crisis event occurs, the evaluation and judgment of the external surrounding environment and the mistakes of the external corporation will affect the emotional response of employees, and the emotional response will not only affect the physical and mental health, but also further affect the employees' communication attitude and behavior (Yu et al., 2021). The outbreak of an external financial crisis increases the corporate uncertainty and ambiguity in the industrial sector, and even causes the managers to subjectively rationalize the fuzzy information. In an external financial crisis characterized by obstructed information and negative emotions, rumors are spread to a certain extent driven by negative emotions (Tse and Bond, 2021), which may cause corporate managers and employees to have more serious anxiety and frustration. Although it is a normal phenomenon that negative events cause negative emotions, the evolution process and direction of negative emotions in corporations are worth pondering. In the group, negative emotions are constantly amplified in the development and evolution of events, and this anxiety has a typical “resistance” (Wang et al., 2021). If it is not channeled and resolved in a timely and effective manner, it may hinder future corporate development planning. Fast and instant response is the basic requirement for corporate development in this era, and the external financial crisis subjects industrial competition and corporate business to great changes. Alarmed by the crisis warning from external enterprises to some extent, the managers carefully examine the problems existing in their enterprise and have a clear and rational understanding of the actual condition, in order to timely reflect and reduce the possibility of a financial crisis. The present study argues that state anxiety strengthens the rational cognition and perceptual moods and proposes the following hypotheses:

*Hypothesis 5: State anxiety mediates the relationship between the operational crisis of external enterprises and management stress*.
*Hypothesis 6: State anxiety mediates the relationship between debt crisis of external enterprises and management stress*.
*Hypothesis 7: State anxiety mediates the relationship between the growth crisis of external enterprises and management stress*.

### Moderating Role of Financial Warning Capability

A notable managerial practice shows that making accurate warnings and preventing corporate financial crisis is an important part of corporate management, and it is the prerequisite for the management to adjust managerial strategies, investment decisions, and financial policies, and to protect the interests of investors and creditors (Broz and Ridzak, 2017). The ultimate value of the financial warning system is in the timeliness and effectiveness of the warning information to let the decision-makers and managers take corresponding timely countermeasures (Jemović and Marinković, 2021). High-level financial warning capability cannot be bought and sold through market transactions like other production factors, that is, they are non-tradable (Samitas et al., 2020), and it is easy to build high-quality continuous barriers to competition. Therefore, the impact of external financial crisis events is similar to other public crisis events (Bergmann and Müller, 2021), which expose the public to uncontrollable potential threats and generate negative emotions such as anxiety in their hearts (Wang et al., 2019). These emotions will further affect public cognition and behavior, and their effects will gradually accumulate (Bussiere and Fratzscher, 2006). Tong (2020) points out that the financial distress prediction that is difficult to imitate is the core competitiveness that corporate managers gradually cultivate and enables them to quickly adapt to and utilize the changes in the long and complicated process.

Overall, the financial warning can effectively monitor the changing trend of corporate financial situation to predict the financial crisis in advance (Xu et al., 2015), make an in-depth analysis of the root causes of the crisis, and assist corporate managers to take effective measures to prevent the crisis, and realize sustainable, stable, and healthy corporate development. The hidden crises in all aspects of the enterprise are the easiest to be disclosed through financial indicators, and a financial warning is the best entry point and the top priority for enterprises to carry out comprehensive crisis management. By analyzing the effectiveness of the financial warning system, Wang and Li (2021) indicate that although the financial warning is an indispensable part of the financial management system, not all the enterprises that are carrying out financial warning work can consciously develop this ability. An internal operation mechanism that is scattered, incomplete, and even lacking important and key links will not only make the financial warning system not function normally, but also cause problems to the financial warning, such as sending out wrong warning signals (Sarlin and Von Schweinitz, 2021), and harm corporate operation and management. The relevant managers who have the financial warning capability can take targeted response plans to solve the existing problems, avoid the gradual deterioration of the corporate financial situation and operating results, and ensure that the production and operation are in an orderly correction and on the right track (He et al., 2019).

In general, most of the research on corporate financial warning management stay at the level of establishing a financial warning system, while there is a lack of organic connections between various components. Besides, it is difficult to form a set of financial warning capability in the true sense (Flink and Chen, 2021). The construction of the financial warning system shall not be separated from other parts of the corporate management system as its single and isolated design and formulation will damage the prediction effect of the probability of financial crises (Helfat and Peteraf, 2015). A complete set of decision-making and enforcement mechanisms is largely the result of the combination of corporate governance and decision-making (Kim and Ko, 2020). Accordingly, the present study puts forward the following hypothesis:

*Hypothesis 8: Financial Warning Capabilities Moderate the Relationship Between the Financial Crisis and Management Stress*.

**Figure 1:**
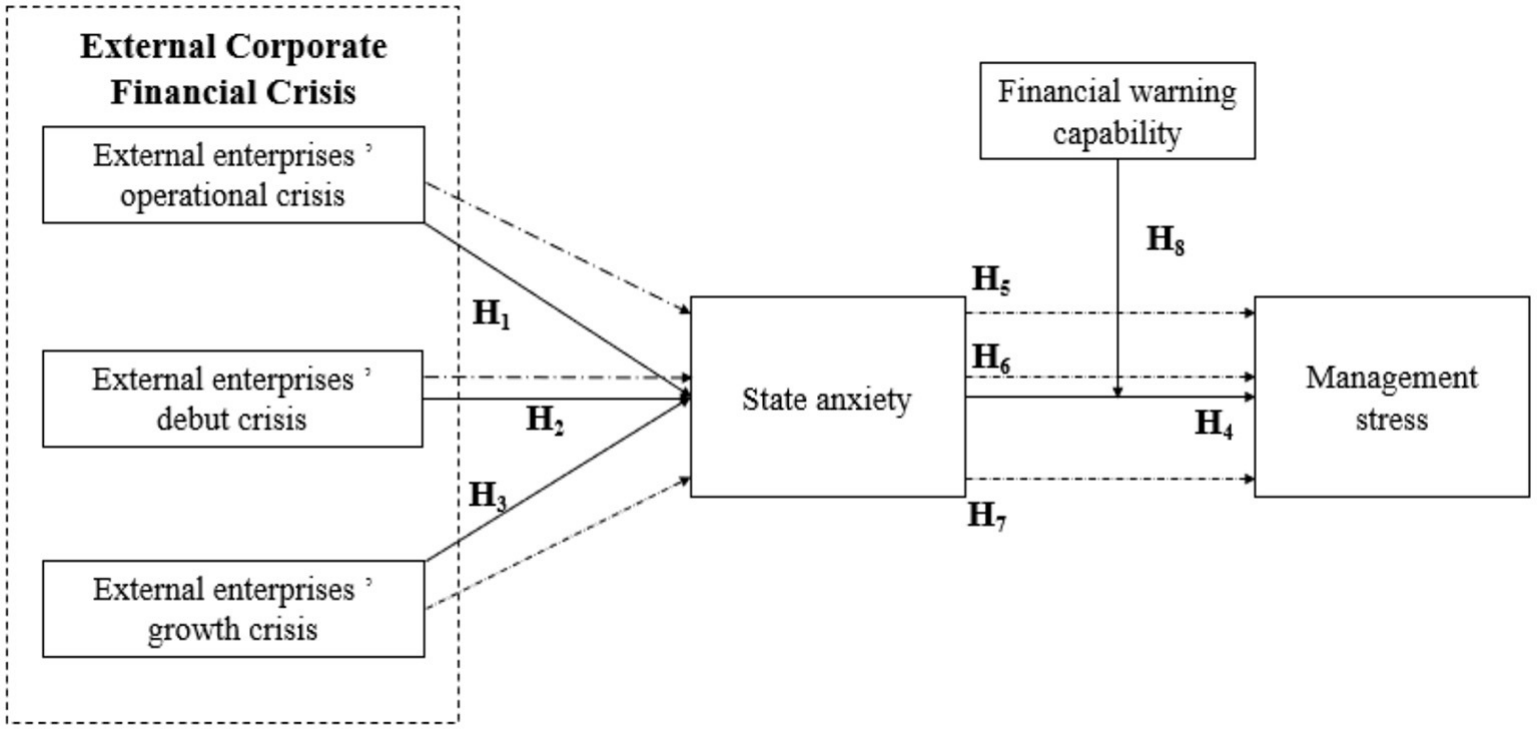
Research framework.

The research framework of this study is shown in Figure 1. The main hypothesis aims to explore the effects of the external corporate financial crisis on the management stress of employees in the financial industry. The empirical analysis reveals that external financial crisis has significant effects on state anxiety, in particular, external corporate operating crisis, debt crisis and growth crisis moderate the relationship between state anxiety and pressure management, and the mediating the relationship between the external financial crisis and pressure management.

## Methodology

### Data Collection

Based on the context requirements of the research objectives, this paper selected senior managers and employees engaged in financial affairs as the subjects, among them, the sample comes from the service and the manufacturing industry. The industries with relatively more distribution are the Internet, hardware manufacturing, consulting and education training, wholesale, and retail, with relatively wide coverage and a certain scientific and representative nature. We sent out questionnaires offline and online from September to December 2020, and altogether collected 376 questionnaires within 3 months, including 347 effective questionnaires acquiring a 92.29% effective rate. According to the statistical characteristics of the questionnaires (see Table 1), the gender ratio is nearly 1:1, with 180 men accounting for 51.87%; the subjects aged 31–45 account for the largest proportion, namely, 50.43%; in terms of corporate types, private enterprises account for 53.89%, followed by state-owned enterprises accounting for 26.80%; the subjects having worked for 5–10 years account for 39.48%, followed by for more than 10 years accounting for 32.85%; the subjects holding a bachelor's degree and above account for 60.81% and those with a master's degree accounting for 21.32%.

**Table 1:** Descriptive statistics.

**Category**	**Demographics**	**Quantity**	**Proportion (%)**
Gender	Male	180	51.87
	Female	167	48.13
Educational background	High school and below	21	6.06
	Junior college	41	11.81
	Undergraduate	211	60.81
	Postgraduate and above	74	21.32
Age	Aged below 30	84	24.21
	Aged 31-45	175	50.43
	Aged above 45	88	25.36
Career experiences	Less than 3 years	20	5.76
	3-5 years	76	21.91
	5-10 years	137	39.48
	More than 10 years	114	32.85
Corporate type	State-owned or collective enterprises	93	26.80
	Private enterprises	187	53.89
	Foreign- funded enterprise (joint venture or sole proprietorship)	67	19.31
Job post	Corporate senior mangers	78	22.48
	Financial staff	269	77.52

*The data comes from a questionnaire survey of 347 financial personnel*.

### Analysis Tools

The questionnaire of this study includes two parts, individual public traits, and public cognition. Referring to the relatively mature scale in previous studies and setting the measurement scale in combination with the research topic. The questionnaire adopts the five-point Likert scale following the previous literature (Li and Katsumata, 2020; Wang and Yi, 2020; Yi et al., 2020), with four theories including the external financial crisis, state anxiety, stress management, and financial risk warning. Three indicators of the external financial crisis are measured using models established by Altman et al. (1977) and Holzhauer et al. (2016), and the respondents are asked to recall financial crisis-related experiences and indicate the level of recognition (e. g., excessive debt stress from external enterprises severely affects the financial position and operational management of the business), and the Cronbach's α value of the operational crisis, debt crisis, and growth crisis of external enterprises is 0.905, 0.885, and 0.887 respectively. State anxiety is measured by combining the STAI scale and SSAI, and the respondents are asked to ponder over psychological and emotional problems when experiencing an external financial crisis, for example, the financial crisis of external enterprises brings negative emotions, such as severe panic and annoyance (Spielberger, 1972; Marteau and Bekker, 1992; Spielberger and Reheiser, 2009), and the Cronbach's α value of state anxiety is 0.885. The management stress was measured by combining the scales proposed by Williams and Cooper (1998) and Cavanaugh et al. (2000). The respondents are asked to recall management issues at the time of an external financial crisis, for example, stress can be translated into internal values and motivation through necessary measures to improve organizational performance), and the Cronbach's α value of the management stress is 0.881. The predicting financial distress is measured by combining the index system of Altman (2013), the respondents are asked to ponder over the usefulness and feasibility of the financial warning system for handling the financial crises (e. g., predicting financial distress can effectively reduce the incidence of the financial crisis), and the Cronbach's α value of the predicting financial distress is 0.891. Besides, SPSS26.0 software is used for the validity test and the KMO value of the overall scale is 0.872 (>0.8). The results show that the reliability and validity of the scales are quite good, indicating there exists reliability of internal consistency among the variables. In this study, structural equation modeling (SEM) was conducted by SPSS.

### Data Analysis

This study conducted a confirmatory factor analysis (CFA), and the results are shown in Table 2, indicating that the standard loading of each factor is between 0.778 and 0.885, all > 0.7; the composite reliability is between 0.901 and 0.921, all > 0.7; and the average variance extracted is between 0.660 and 0.767, all > 0.6. The results of the confirmatory factor analysis in this study all met the criteria, with good convergent validity.

**Table 2:** Confirmatory factor analysis.

**Construct**	**Item**	***p***	***STD*.**	***SMC***	***CR***	***AVE***
OC	OC1		0.868	0.696	0.921	0.700
	OC2	0.000	0.861	0.725		
	OC3	0.000	0.785	0.584		
	OC4	0.000	0.832	0.651		
	OC5	0.000	0.836	0.637		
DC	DC1		0.778	0.552	0.906	0.660
	DC2	0.000	0.833	0.646		
	DC3	0.000	0.793	0.56		
	DC4	0.000	0.844	0.642		
	DC5	0.000	0.811	0.645		
GC	GC1		0.842	0.629	0.914	0.726
	GC2	0.000	0.866	0.689		
	GC3	0.000	0.829	0.598		
	GC4	0.000	0.871	0.737		
SA	SA1		0.813	0.264	0.901	0.695
	SA2	0.000	0.857	0.232		
	SA3	0.000	0.851	0.193		
	SA4	0.000	0.813	0.288		
FC	FC1		0.867	0.599	0.905	0.705
	FC2	0.000	0.849	0.72		
	FC3	0.000	0.823	0.632		
	FC4	0.000	0.818	0.759		
MS	MS1		0.884	0.704	0.908	0.767
	MS2	0.000	0.885	0.722		
	MS3	0.000	0.858	0.695		

The data comes from a questionnaire survey of 347 financial personnel and the items refer to existing measurement scales, such as STAI and SSAI. OC, operational crisis; DC, debut crisis; GC, growth crisis; SA, state anxiety; FC, financial warning capability; MS, management stress; and ***p < 0.001.

The discriminant validity of the measurement model is analyzed and the results show a significant correlation among the variables (*p* < 0.01), the absolute values of the correlation coefficient are all <0.5, which are all smaller than the corresponding AVE square root, indicating that the discriminant validity of the scale data is ideal (Table 3).

**Table 3:** Discriminant validity.

	**Mean**	**SD**	**OC**	**DC**	**GC**	**SA**	**FC**	**MS**
OC	3.78	1.06	**(0.700)**					
DC	3.89	1.004	0.251^**^	**(0.660)**				
GC	3.84	1.022	0.103^**^	0.163^**^	**(0.726)**			
SA	3.75	1.106	0.305^**^	0.270^**^	0.235^**^	**(0.695)**		
FC	3.71	1.059	0.158^**^	0.228^**^	0.235^**^	0.271^**^	**(0.705)**	
MS	3.66	1.103	0.199^**^	0.290^**^	0.279^**^	0.278^**^	0.274^**^	**(0.767)**
AVE square root			0.837	0.812	0.852	0.834	0.840	0.876

** Significantly correlated at the 0.01 level (two-sided); the diagonal values (bold) are AVE (Average Variance Extracted). ***p < 0.001; **p < 0.01; *p < 0.05.

**Figure 2:**
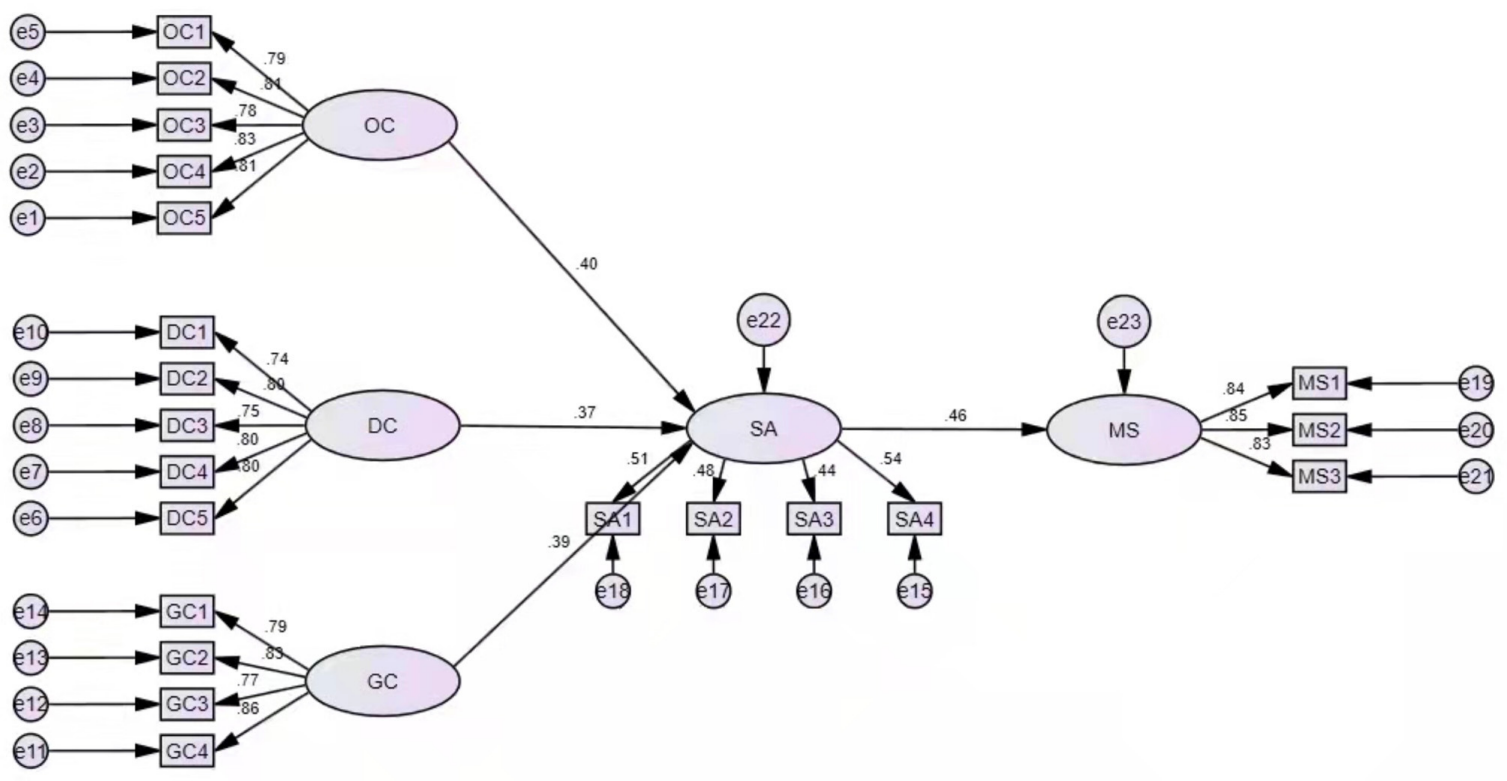
Structure model.

A modified mediating model has been set up in this study (see Figure 2) to further verify the research hypotheses presented above. As shown in Table 4, the results indicate that the operational crisis of external enterprises has a significant impact on state anxiety, the path coefficient is equal to 0.402, CR = 4.235, and *p* < 0.05; The debt crisis of external enterprises has a significant impact on state anxiety, the path coefficient is equal to 0.365, CR = 4.083, and *p* < 0.05; the growth crisis of external enterprises has a significant impact on state anxiety, the path coefficient is equal to 0.390, CR = 4.546, and *p* < 0.05, thus Hypothesis 1, 2, and 3 are all verified; state anxiety has a significant impact on management pressure, the path coefficient is equal to 0.458, CR = 4.955, *p* < 0.05, thus Hypothesis 4 is verified.

**Table 4:** Hypothesis testing.

**DV**	**IV**	**Unstd**	**S.E**.	**C.R**.	***p***	**Std**.	***R* ^2^**	**Result**
SA	OC	0.236	0.056	4.235	^***^	0.402	0.447	Supported
	DC	0.237	0.058	4.083	^***^	0.365		Supported
	GC	0.248	0.054	4.546	^***^	0.390		Supported
MS	SA	0.773	0.156	4.955	^***^	0.458	0.210	Supported

*p < 0.05; **p < 0.01;

*** *p < 0.001*.

The goodness-of-fit testing was conducted to affirm the structural model, and the results are shown in Table 5. Comparing the suggested values of each index with the actual results of this study, each index meets the basic standards. Overall, the fitting indices of this study meet the requirements, indicating that the structural model of this study is acceptable, the goodness-of-fit is satisfactory, and the study results are acceptable.

**Table 5:** Goodness-of-fit of the structural model.

**Index type**	**Fitting index**	**Suggested value**	**Actual value**	**Fitting effect**
Absolute fitting index	CMIN/DF	1–3	1.469	Ideal
	GFI	>0.9	0.933	Ideal
	RMSEA	<0.05	0.037	Ideal
Value-added fitting index	CFI	>0.9	0.98	Ideal
	IFI	>0.9	0.98	Ideal
	TLI	>0.9	0.976	Ideal
Simple fitting index	PGFI	>0.5	0.719	Ideal
	PCFI	>0.5	0.831	Ideal
	PNFI	>0.5	0.797	Ideal

To further ensure the reliability of the research results and examine the mediating effect of state anxiety, the Bootstrap test has been performed with 5,000 samples, and a 95% confidence interval has been selected. The results are shown in Table 6. The total effect is significant (*r* = 0.402, SE = 0.120, *z* = 3.350, *p* < 0.05), and at the 95% confidence interval, the confidence interval generated with the Bias-corrected estimation method is (0.119, 0.425); the indirect effect is significant (*r* = 0.184, SE = 0.050, *z* = 3.680, *p* < 0.05), and at the 95% confidence interval, the confidence interval generated with the Bias-corrected estimation method is (0.034, 0.159); the direct effect is not significant (*r* = 0.218, SE = 0.120, *z* = 1.817, *p* > 0.05), and thus supported the mediating effect of state anxiety on the operational crisis and management stress of external enterprises, and it is fully mediating, and H5 is established. Similarly, H6 and H7 are all verified and are fully mediating.

**Table 6:** Empirical testing of mediating effects.

**Path**	**Point estimate**	**Coefficients**			**Bootstrap**			
					**Bias-corrected 95%**		**Percentile 95%**	
		**SE**	***Z*-value**	***p*-value**	**Lower**	**Upper**	**Lower**	**Upper**
Overall effect OC → MS	0.402	0.120	3.350	0.001	0.119	0.425	0.117	0.423
Indirect effect OC → SA → MS	0.184	0.050	3.680	0.000	0.034	0.159	0.031	0.152
Direct effect OC → MS	0.218	0.120	1.817	0.069	0.020	0.191	0.020	0.190
Overall effect DC → MS	0.365	0.117	3.120	0.002	0.075	0.370	0.050	0.360
Indirect effect DC → SA → MS	0.167	0.055	3.036	0.002	0.022	0.138	0.018	0.130
Direct effect DC → MS	0.198	0.117	1.692	0.091	0.013	0.167	0.011	0.162
Overall effect GC → MS	0.390	0.114	3.421	0.001	0.085	0.379	0.078	0.373
Indirect effect GC → SA → MS	0.179	0.065	2.754	0.006	0.020	0.154	0.018	0.145
Direct effect GC → MS	0.211	0.114	1.851	0.064	0.015	0.171	0.013	0.168

**Figure 3:**
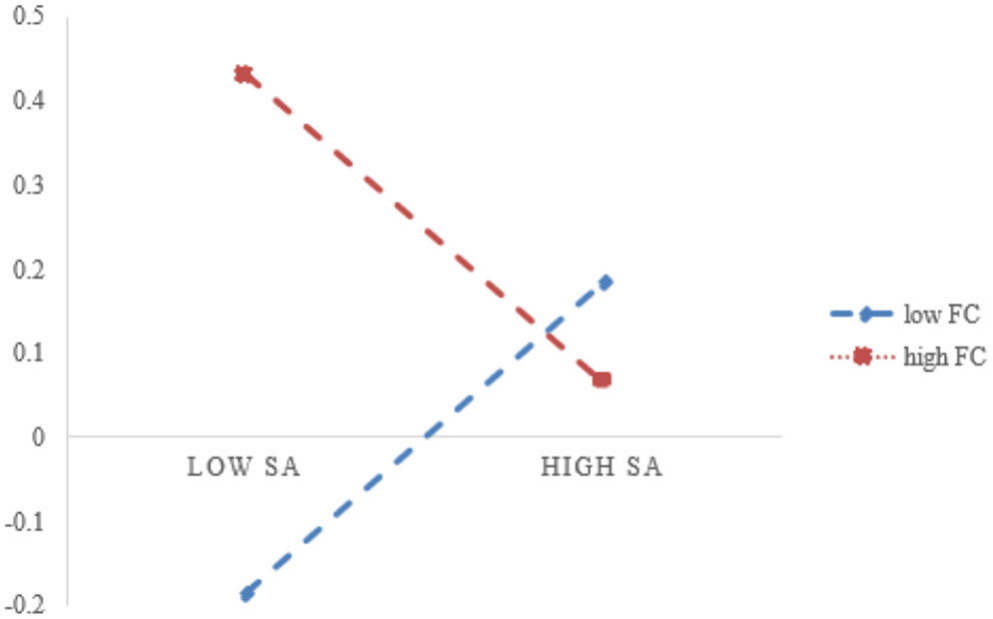
Mediating role of financial warning capability.

In this study, the independent and moderating variables are decentralized, and the interaction term of state anxiety and financial warning capabilities is constructed and validated together with the original model to test the moderating effect of predicting financial distress, as shown in Table 7. After being corrected, the interaction coefficient of state anxiety and predicting financial distress is −0.370, *p* < 0.05, indicating that the financial distress prediction has a significant negative mediating effect on state anxiety and pressure management. However, as shown in Figure 3, with low financial distress prediction, state anxiety has more significant effects on pressure management, thus Hypothesis 8 is verified.

**Table 7:** Moderating effect testing.

	**Non-standardized coefficient**	**Standardized coefficient**	**S.E**.	**C.R**.	***P***
SA → MS	0.200	0.186	0.067	2.998	0.003
FC → MS	0.270	0.247	0.069	3.897	^***^
Int → MS	−0.052	−0.370	0.056	−0.921	0.037

*Interaction term of SA and MS*.

*** *p < 0.001*.

## Discussion and Implications

This study aims to construct the conceptual model of the financial crisis of the external enterprise affecting the management stress and explore how it affects the state anxiety under the mediation of the financial warning and further affects the management stress of employees in the financial industry. Through theoretical exploration and empirical analysis, the following conclusions are drawn.

First, the external financial crisis has a significant positive impact on state anxiety. Specifically, the operational crisis, debt crisis, and growth crisis of external enterprises all have a significant impact on state anxiety, and the degree of impact is relatively similar. The research results are consistent with those of Onsongo et al. (2020) and Ruan and Liu (2021), conforming the actual market circumstance. Corporate financial managers tend to pay attention to the management status of external enterprises to review their management ability and examine their market competitiveness, and the enterprises may need to re-examine the strategies already formulated and adjust the strategic direction in time to make up for the side effects caused by the financial crisis.

Second, state anxiety has a significant positive effect on pressure management. The results are consistent with those of Norris et al. (2020) and Hannah et al. (2021), conforming to the real logic. Crises will break the psychological balance of the managers, cause state anxiety and changes in their cognitive response process, resulting in the decline of thinking and judgment ability, unable to effectively resolve environmental interference, and leading to frustration in adaptation to the market circumstances and pressure management.

Third, state anxiety plays a mediating role in the impact of the external financial crisis on pressure management. The research results are the same as those of Tse and Bond (2021) and Wang et al. (2021), which are in line with the corporate actual situation. The financial crisis is not a static and independent incident, but an integral and dynamic process. State anxiety itself will occupy more corporate cognitive resources, which will affect the input of the employees and increase the management stress of managers.

Fourth, under different financial warning capabilities, there are significant differences in the impact of the financial crisis on pressure management. The research findings are consistent with those of scholars including Tong (2020) and Wang and Li (2021), conforming to the real context. While the financial crisis of external enterprises brings stress to the financial managers, it has a prominent warning and enlightening function, and enhancing the financial distress prediction is bound to reverse the impact of the financial crisis of external enterprises.

The external financial crisis events have shown a certain performance of the times. The financial crisis is not a static and independent event, but an overall and dynamic development process. This study can better understand the public's perception and how managers respond to crises and reduce management pressure. The results of the survey sample provide strong insights for our study. Therefore, this study is of great significance to the research and events of the applied psychology of corporate management.

### Theoretical Implications

Our findings have made important theoretical implications. Although the past research results show that there is a significant positive relationship between the financial crisis and management pressure (Huang and Yen, 2019), however, related research in management and psychology is relatively narrow, even if the topic of the financial crisis has been studied for many years, studying how the emotions and behaviors affecting financial personnel from the external crisis perspective are also known. Even people will argue that we have a good understanding of the financial crisis, which can prevent or even avoid it. However, such a statement is likely to be wrong. Our research shows that the external enterprise crisis will release signals, enabling financial personnel from other companies in the industry to be highly nervous and anxious, which will affect their management decisions.

This study provides a convincing research perspective for the research on the financial crisis, covering internal control and the external competitors. First, this study contributes to the literature research on the external financial crisis affecting management stress by identifying status anxiety as an important influencing factor of pressure management. The results show that state anxiety deserves the attention of researchers and practitioners, which is an effective way to reduce management stress and “restart” employees' working enthusiasm and efficiency during the special period of accelerating economic globalization. It is shown in this paper that a financial crisis in external enterprises will bring different levels of anxiety to financial managers and related employees and increase the management's operation and managerial pressure.

Second, this paper proposes that state anxiety is the mediating factor for the impact of the external financial crisis on pressure management. State anxiety brings certain psychological expectations to the management to use the existing corporate resources to reduce the emergence of negative emotions and inject positive energy into the employees and enterprises. Previous studies focused on exploring the relationship between state anxiety and crisis (Loriette et al., 2019), state anxiety and management stress (Wang X. et al., 2020), and crisis and management stress (Turgaeva et al., 2020). The present study defines the mediating role of state anxiety in external financial crisis and pressure management, which is a beneficial supplement to previous studies.

Additionally, this paper demonstrates the moderating role of financial distress prediction in the impact of state anxiety on pressure management. If the enterprise has a high financial distress prediction, it will effectively supervise the changing trend of its financial situation when facing the crises of others, and predict the financial crisis in advance, which will help to relieve the stress of the management facing financial crises and enhance its market competitiveness. Moreover, many studies have focused on exploring the direct role of financial distress prediction (Wu et al., 2021), and this paper is a further extension of this series of studies.

### Managerial Implications

In line with regularity exploration, this paper puts forward the following practical implications for future corporate financial management. First, senior managers need to pay attention to the prospects of the industrial sector and external market competition. Enterprises in the market competition strive for survival in the present time and development in the future, so they need to fully grasp the market competitive environment. In the “industrial scenario” analysis, starting with the competitive intensity in the global prediction “scenario” (Gauger et al., 2021), the enterprises should accurately make expected positioning and consider refining the factors affecting the uncertainty, source of competitive advantage, and possible actions of competitors by employing analysis. The enterprises should consider the competitive intensity of the industry as well as its competitive status in the industry. It should be noted that enterprises in the same industry will not only have conflicts in their products and profits, but also form a competitive relationship in terms of obtaining external financing and consumers. To better understand the external risks of industrial competition, it is necessary to clarify the transmission process of strengthening and adjusting the corporate competitive status, accurately study and judge the financial crisis and possible harms and make timely and forward-looking warnings.

Second, the enterprises need to improve their financial distress prediction and internal control system. The financial warning system has its features, which can use the data format to set up the crisis warning mechanism and have insight into the risks existing in the internal and external corporate environment. Organizations can prevent crises with two approaches. One is the corporate warning system. The enterprises should set up a comprehensive warning mechanism, and set up risk warning indicators for material production, sales, and other related departments taking into account profitability, solvency, economic efficiency, corporate development potential, financial flexibility, profitability, debt management, and other financial risks (Kuang et al., 2019), and set the warning values to which the indicators belong in line with the corporate characteristics. Second, this study advances the literature on financial warning mechanisms. The warning mechanism should be set up in line with financial indicators, and thus a tight system is expected to be formed to effectively prevent the enterprise from a financial crisis.

In addition, pertinent employees need to adjust their anxieties and control the corporate pressure management. As financial crises in an individual enterprise or the same industry occur, the negative sentiment of the relevant employee increases, the market becomes vulnerable and sensitive, and the butterfly effect caused by the uncertainty of the financial crisis may lead to huge market volatility and corporate turbulence. The enterprise should dynamically grasp the anxiety of employees, customize psychological checkups, and provide a stable and formal communication platform and media as well as emotional or instrumental support for employees, which can relieve the management stress induced by anxieties.

As a financial manager, controlling pressure management is not eliminating the pressure source, but mastering the right work feedback method, abandoning the subjective decision, such as experience and “taking the head”, enhancing the ability of relevant personnel to understand the crisis, analyze the crisis and prevent the crisis. In addition, attaching importance to employee emotional management, enhance employee's mental knowledge reserves to respond to negative emotions and work pressure, and effectively enhance the belongingness. Based on the important position of financial personnel in the prevention of financial crisis, training for financial personnel should not be limited to accounting continuing education, but also improve its quality, master the crisis management theory, and accurately analyze the external environment and changes. It is possible to make prevention measures to reduce the possibility of a financial crisis.

### Limitations and Future Research

In this paper, the mechanism of management stress induced by the financial crisis of external organizations are identified and further verified the generation law of the crisis anxiety of others. Our work has some limitations and provides future research directions. First, in the current research, the sample data is limited to China's region, and the sample coverage still has certain limitations. Whether the research conclusions are universal needs to be further investigated; second, as the sample data are questionnaire-based, the data source is not diverse. Future studies can further infer the relationship between variables with corresponding respective semi-structural interviews and experimental methods. Despite the mediation role of state anxiety being explored, the negative impact is also investigated. Whether state anxiety will lead to active behavior and performance is still unclear, future research can be conducted to test the role of state anxiety in different fields and different contexts. Finally, the current research results are more prominent in specialized industries (non-monopoly industries), such as accounting and auditing.

## Data Availability Statement

The raw data supporting the conclusions of this article will be made available by the authors, without undue reservation.

## Ethics Statement

The studies involving human participants were reviewed and approved by Shenzhen University. The patients/participants provided their written informed consent to participate in this study. Written informed consent was obtained from the individual(s) for the publication of any potentially identifiable images or data included in this article.

## Author Contributions

BL contributed to the empirical work, the analysis of the results, and the writing of the first draft. JZ significantly supported the overall work of this article. BW and YW supported the work of BL and JZ. FS contributed to the overall quality, supervised the literature organization, and empirical work. All authors discussed the results and commented on the manuscript.

## Funding

This study was supported by National Natural Science Foundation of China (No. 71772128), Social Science Planning General Project in Jiangxi Province (No. 21JY17), and Social Science Young Project in Jiangxi Province (No. 21JY54).

## Conflict of Interest

The authors declare that the research was conducted in the absence of any commercial or financial relationships that could be construed as a potential conflict of interest.

## Publisher's Note

All claims expressed in this article are solely those of the authors and do not necessarily represent those of their affiliated organizations, or those of the publisher, the editors and the reviewers. Any product that may be evaluated in this article, or claim that may be made by its manufacturer, is not guaranteed or endorsed by the publisher.
